# Persistent Adaptation Policy and National Food Self-Sufficiency: Within- and Between-Country Evidence from a Global Panel

**DOI:** 10.3390/foods15193492

**Published:** 2026-09-29

**Authors:** Sagit Barel-Shaked, Erez Buda

**Affiliations:** 1 Department of Economics, Western Galilee College, Akko 2412101, Israel; 2 Department of Management, Western Galilee College, Akko 2412101, Israel; erezb@wgalil.ac.il

**Keywords:** food self-sufficiency, agricultural adaptation policy, climate finance, climate governance, panel data, Mundlak decomposition, within–between decomposition

## Abstract

Climate change affects domestic agricultural production, but national food self-sufficiency also depends on institutional and structural conditions that operate over different time horizons. This study examines these relationships using a balanced panel of 101 countries observed annually from 2012 to 2022 (1111 country-year observations). Food self-sufficiency is measured with the Food Self-Sufficiency Ratio (SSR), with SSR > 85% as the primary binary outcome. A pooled logistic regression with a within–between decomposition following the Mundlak approach separates persistent between-country differences from within-country changes, with country-clustered standard errors. The most consistent finding is a positive between-country association between long-run agricultural adaptation-policy status and high self-sufficiency (OR = 6.676, 95% CI: 1.502–29.668), whereas within-country changes in policy status are not statistically significant. This association reflects a persistent cross-country difference and should not be interpreted as a causal short-run effect of policy adoption. Within-country increases in climate-finance scores are negatively associated with the 85% threshold, but this result is sensitive to the outcome definition. Sensitivity analyses using 75%, 80%, and 90% thresholds show that the adaptation-policy association remains positive across all specifications and is the most stable result overall, although it is no longer statistically significant at the 90% threshold. The findings underscore the importance of distinguishing persistent institutional conditions from short-run policy changes when analyzing national food self-sufficiency.

## 1. Introduction

Climate change is reshaping the conditions under which national food systems operate. Rising temperatures, drought, flooding, altered precipitation patterns, soil degradation, and increasingly frequent extreme events affect agricultural productivity directly, while disruptions to transport, storage, markets, and supply chains transmit climatic shocks far beyond the farm sector [1,2,3,4]. Meta-analytic evidence shows that warming reduces yields of major staple crops, while global modeling indicates that climate change, population growth, and land-use pressures may jointly intensify future risks to food availability and undernourishment [2,3,4,5]. Climate shocks can also reduce irrigation potential and soil moisture, damage infrastructure, impair livestock production, raise food prices, and erode household incomes, thereby linking biophysical exposure to broader food-system vulnerability [4,6,7].

The effects of climate stress, however, are not uniform across countries and are not determined by exposure alone. Countries facing comparable environmental pressures may differ markedly in their capacity to sustain domestic agricultural production because climatic risk interacts with productive resources, economic structure, institutional capacity, and public policy. Adaptation may involve improved irrigation and water management, sustainable land management, climate-smart agriculture, diversification, early-warning systems, and technological innovation [3,8,9]. Yet a recurring conclusion in the adaptation literature is that technical measures are rarely sufficient on their own. Their adoption, coordination, and persistence depend on access to finance, institutional support, policy coherence, risk-management capacity, and the ability to coordinate action across levels of government [6,10,11]. Farm-level evidence points in the same direction: access to credit, training, farmer organizations, and institutional support shapes both the adoption and effectiveness of adaptation strategies [12]. These findings suggest that climatic exposure and institutional capacity are best understood as interdependent dimensions of food-system resilience.

Food self-sufficiency provides a specific lens for examining the extent to which national food supply relies on domestic production. It should not be equated with food security: countries may achieve food security while relying substantially on imports, whereas high domestic production does not necessarily ensure affordability, nutritional adequacy, stability, or equitable access [13,14]. Accordingly, SSR is treated here as a distinct characteristic of national food systems rather than as a comprehensive measure of food security or a normative objective of food autarky [13,15]. This distinction is particularly relevant under climate change because climatic pressures may constrain domestic agricultural production, while international trade can both buffer localized production shocks and expose countries to external market and supply disruptions.

Existing research provides strong evidence on individual components of this problem. Climate-impact studies document the adverse effects of warming and extreme events on crop yields, water resources, soils, and agricultural production [2,3,7]. Adaptation studies identify practices that can reduce vulnerability and improve agricultural resilience [8,12]. Related literature emphasizes governance, policy coherence, institutional coordination, and financing as enabling conditions for effective adaptation [6,10,16,17]. At the same time, research on self-sufficiency emphasizes the importance of trade, economic structure, and domestic productive capacity [13,15]. What is less well understood is how these climatic, institutional, policy, and structural dimensions jointly relate to high national food self-sufficiency when their temporal characteristics differ substantially.

That temporal distinction is especially important for institutional and policy variables. Many governance indicators used in cross-country research are highly persistent and change only infrequently. A country that has maintained an agricultural adaptation framework over much of a decade represents a different institutional condition from a country that adopts such a framework in a particular year. Likewise, persistent differences in climate-related development assistance may reflect structural vulnerability, long-run adaptation needs, or enduring donor–recipient relationships, whereas year-to-year increases may respond to emerging risks, project cycles, or acute financing needs. Treating these forms of variation as equivalent can blur the distinction between enduring national characteristics and short-run changes. In practical terms, comparing two countries with different long-run policy profiles is not the same empirical question as asking whether a change within one country is associated with a contemporaneous change in self-sufficiency.

The same logic applies across the broader set of factors examined in this study. Projected flood exposure represents a persistent environmental constraint that can damage crops, infrastructure, storage, and transport systems. Agricultural land provides structural productive potential, although land availability alone cannot guarantee output because productivity also depends on water, soil quality, technology, crop choice, and management practices [3,18,19]. Economic development is theoretically ambiguous: higher income can strengthen agricultural productivity through infrastructure, technology, and institutional capacity, but it can also reduce the need for domestic production by increasing purchasing power and access to international food markets [13,15]. The governance indicators capture distinct but complementary mechanisms. National agricultural adaptation policy reflects whether adaptation for agriculture is embedded in a current national climate strategy; commitment to managing exposure reflects the incorporation of agriculture-related climate exposure and natural-resource management in national climate commitments; risk-management coordination captures alignment between local disaster-risk-reduction strategies and national frameworks; and early-warning and climate-smart-agriculture commitment reflects formal preparedness and anticipatory adaptation. Climate-finance flows add a further dimension because they may represent both adaptive capacity and underlying need: additional resources can support resilience, but higher inflows may also be directed toward countries facing greater vulnerability or institutional constraints.

These mechanisms imply that between-country and within-country associations need not have the same magnitude or even the same direction. Persistent cross-country differences may reflect accumulated institutional capacity, policy continuity, exposure, and structural conditions that operate over long horizons. Within-country changes, by contrast, capture departures from a country’s own long-run level and may reflect policy adoption, temporary financing shifts, or responses to emerging risk. Formal adaptation policies may also require time to become embedded, implemented, and translated into production outcomes, so contemporaneous policy change should not automatically be expected to produce an immediate change in self-sufficiency. Conversely, short-run increases in climate finance or risk-management activity may be reactive, occurring precisely when vulnerability is rising. Distinguishing these two temporal dimensions is therefore not merely a statistical refinement; it is central to the substantive interpretation of how national adaptation and institutional conditions relate to domestic food-production capacity.

This study addresses that gap using a balanced panel of 101 countries observed annually from 2012 to 2022. It integrates national food-production and trade data with indicators of flood exposure, climate-related development assistance, commitment to managing agricultural climate exposure, disaster-risk-management coordination, early-warning and climate-smart-agriculture commitment, national agricultural adaptation policy, GDP per capita, and agricultural land availability. Unlike our previous study [15], which developed a cross-sectional typology of food self-sufficiency among OECD countries, the present study examines a global longitudinal panel of 101 countries and addresses whether climatic, institutional, policy, and structural associations with food self-sufficiency reflect persistent differences between countries or changes occurring within countries over time. The empirical analysis therefore distinguishes between-country and within-country variation through a within–between specification based on the Mundlak decomposition [20]. This approach is particularly well suited to a dataset in which some institutional variables are highly persistent while others exhibit meaningful variation over time, because it allows long-run country characteristics and shorter-run deviations to be examined simultaneously.

Based on the mechanisms outlined above, we expect persistent flood exposure to be negatively associated with high food self-sufficiency, whereas the persistent presence of formal agricultural adaptation policy and stronger risk-management capacity are expected to be positively associated with it. For policy indicators, the between-country associations are expected to be more pronounced than contemporaneous within-country associations because formal institutional changes may require time before they are reflected in agricultural production. The expected associations for climate finance and GDP are less directional. Climate finance may support adaptive capacity, but higher flows may also be targeted toward countries or periods of greater vulnerability; similarly, higher income may strengthen domestic productive capacity while simultaneously facilitating greater reliance on international food markets. These competing mechanisms make the sign of their associations an empirical question and provide an additional reason to distinguish persistent between-country differences from within-country changes.

The central research question is therefore: Which climatic, institutional, policy, and structural characteristics are associated with a country’s likelihood of attaining high food self-sufficiency, and to what extent do these associations reflect persistent differences between countries rather than changes within countries over time? High self-sufficiency is evaluated primarily at an SSR threshold of 85%, with alternative thresholds used to assess the sensitivity of the conclusions. The study contributes to the literature in three ways. First, it brings research on climate exposure and agricultural adaptation into direct dialogue with the literature on national food self-sufficiency while preserving the conceptual distinction between self-sufficiency and broader food security. Second, it treats adaptation governance through multiple dimensions of policy commitment, coordination, preparedness, and climate-related finance rather than as a single undifferentiated institutional construct. Third, it explicitly separates persistent cross-country differences from within-country changes, providing a more precise basis for assessing whether high food self-sufficiency is primarily associated with enduring national conditions or with shorter-run shifts in policy, finance, and institutional capacity.

## 2. Materials and Methods

### 2.1. Data, Sample, and Measurement

The final analytical sample comprised a balanced panel of 101 countries observed annually from 2012 to 2022, yielding 1111 country-year observations. Countries were retained when complete and continuous information on the dependent variable and the covariates required for the final specification was available throughout the study period. Requiring complete longitudinal information may limit the representativeness of the analytical sample because data availability may differ systematically across countries. The 101 countries included in the final analytical sample are listed in Supplementary Table S1, and the complete processed country-year analytical dataset is provided in the Supplementary Materials. The dataset integrates indicators from the Global Food Security Index (GFSI) developed by Economist Impact [21] with agricultural and macroeconomic information derived from the Food and Agriculture Organization of the United Nations [22]. The GFSI Sustainability and Adaptation dimension supplies the measures of flood exposure, climate-finance flows, commitment to managing exposure, risk-management coordination, early-warning commitment, and national agricultural adaptation policy. FAO data provides production, import, and export quantities used to construct the Food Self-Sufficiency Ratio (SSR), GDP per capita adjusted for purchasing power parity, and agricultural land [22,23]. The analytical file retains the harmonized GFSI scores used for cross-country comparison. These indicators were obtained directly from Economist Impact’s publicly released GFSI data files without further transformation by the authors. Economist Impact reports the Sustainability and Adaptation category continuously from 2012 to 2022 under its current scoring framework; the organization periodically reviews and revises this framework in consultation with an expert panel (notably in 2017, when the category was introduced, and again in 2022), and published historical values are presented on a current-methodology basis to preserve cross-time comparability. Because indicator-level revision histories are not separately documented by Economist Impact, some residual measurement variation attributable to these periodic reviews cannot be fully ruled out. We further note that temperature rise and drought risk are time-invariant climate-projection inputs (from ND-GAIN and WRI Aqueduct, respectively) rather than annually re-scored items, and that commitment to managing exposure and early-warning commitment show no within-country variation over 2012–2022 in our sample; these four indicators are accordingly entered as between-country characteristics only. Climate-finance flows, agricultural adaptation policy, and risk-management coordination, by contrast, exhibit meaningful within-country change (in 74, 65, and 60 of the 101 countries, respectively) and are entered with both between- and within-country components.

National food self-sufficiency is measured using the standard formula [13,23]:

SSR_it_ = [Production_it_/(Production_it_ + Imports_it_ − Exports_it_)] × 100

Production, import, and export quantities were obtained from the FAOSTAT Food Balance Sheets using Production (element code 5511), Import quantity (element code 5611), and Export quantity (element code 5911), reported in 1000 tonnes. For each country-year, the quantities recorded for the food-item categories retained in the analytical source file were aggregated separately for production, imports, and exports before calculating SSR. This aggregate quantity-based operationalization follows the approach applied in our previous analysis [15]. It should be distinguished from a complete commodity-level food-balance utilization account: stock variation, feed, seed, losses, processing, and other non-food uses are not separately incorporated in the calculation.

The SSR is widely used in agricultural and food-policy research to assess the extent to which domestic production meets apparent domestic food supply [13,23]. In the formula above, SSR is expressed as a percentage. For statistical estimation, the measure is stored in proportional form in the analytical dataset; thus, an SSR of 85% corresponds to 0.85. Food self-sufficiency is conceptually continuous rather than defined by a universally accepted cutoff [13]. The primary dependent variable, SSR_D85, therefore uses 85% as an operational definition of comparatively high self-sufficiency rather than as a natural or universally established boundary. This choice is consistent with the empirical classification applied by Barel-Shaked and Buda [15], in which SSR values of 85% or below represented low self-sufficiency and values above 85% represented higher self-sufficiency categories. Because dichotomizing a continuous measure necessarily entails some loss of information and results may depend on the selected cutoff, the primary 85% specification is complemented by sensitivity analyses using thresholds of 75%, 80%, and 90%.

SSR is interpreted strictly as a measure of reliance on domestic production and not as a comprehensive measure of food security, nutritional adequacy, affordability, or distributional access.

The explanatory variables represent climate exposure, climate-related finance, institutional capacity, policy preparedness, and structural economic conditions. Table 1 provides the definitions, coding, sources, and empirical roles used in estimation. Flooding is the retained climate-exposure indicator in the final specification. Temperature rise and drought were also evaluated as alternative climate-risk measures, but their inclusion did not improve model fit or provide additional explanatory power conditional on the other covariates; they were therefore omitted from the parsimonious final model.

Climate-finance flows, commitment to managing exposure, and risk-management coordination capture financial and institutional dimensions of adaptation. Early-warning commitment and national agricultural adaptation policy are included as binary policy indicators. The logarithm of GDP per capita and the share of agricultural land are included as structural controls.

### 2.2. Empirical Strategy

The temporal behavior of each explanatory variable was evaluated before estimation by separating its variation into between-country and within-country components. For a time-varying predictor X_it_, the country-specific mean, $\bar{X}$_i_, captures persistent differences across countries, whereas the deviation X_it_ − $\bar{X}$_i_ captures the value of the predictor in a given year relative to that country’s own long-run average. This distinction is necessary because several climate and governance indicators vary primarily across countries rather than within countries over time.

The main analysis uses a pooled binomial logistic regression with a within–between decomposition of the time-varying covariates following the Mundlak approach [20]. Country-specific means represent between-country variation, while deviations from those means represent within-country variation. The primary specification does not include a country-level random intercept. Flood exposure, climate-finance flows, risk-management coordination, agricultural adaptation policy, and GDP per capita are represented by both their country means and their within-country deviations. Commitment to managing exposure and early-warning commitment showed no within-country variation and are therefore represented only by their country-level means. Agricultural land displayed measurable within-country variation; however, adding a within-country land component did not improve model fit (likelihood-ratio test *p* = 0.78) and increased AIC from 1244 to 1246 and BIC from 1314 to 1321. Agricultural land is therefore retained as a country-level structural component only.

The hybrid specification is preferable to a conventional fixed-effects logit for the present research question. A fixed-effects logit would identify associations exclusively from within-country changes, omit countries with no change in the binary outcome, and prevent estimation of variables that are constant or nearly constant over time. The within–between approach retains substantively important cross-country information while separately identifying within-country associations for variables that change over time.

$$\mathrm{logit}[P(\mathrm{SSR}\text{\_}D85_{\mathrm{it}}=1)]=\alpha +\beta B\;{\bar{X}}_{i}+\beta W(X_{\mathrm{it}}-{\bar{X}}_{i})+{\mathrm{\gamma P}}_{\mathrm{it}}+{\mathrm{\delta Z}}_{\mathrm{it}}$$

In this expression, $\bar{X}$_i_ denotes country-specific means, between-country components (B), X_it_ − $\bar{X}$_i_ denotes within-country deviations (W), P_it_ denotes the policy indicators, and Z_it_ denotes the structural controls. A between-country coefficient compares countries with different long-run average values of a predictor. A within-country coefficient evaluates whether a year in which a predictor is above or below the same country’s long-run average is associated with a change in the probability of high self-sufficiency. The model was estimated using a binomial logit link with standard errors clustered at the country level to account for within-country dependence and heteroskedasticity across repeated annual observations. Coefficients are reported as log-odds and odds ratios, with 95% confidence intervals calculated from the country-clustered covariance matrix.

### 2.3. Model Selection, Diagnostics, and Sensitivity Analysis

The final specification was selected by jointly considering theoretical relevance, model fit, and parsimony. Average temperature rise and drought were evaluated as additional climate indicators, but their inclusion did not improve fit relative to the reduced specification. A likelihood-ratio comparison of the expanded and reduced models was not statistically significant (χ^2^ = 2.05, df = 2, *p* = 0.36). The reduced model also produced lower information criteria (AIC = 1277 versus 1279; BIC = 1337 versus 1349). Flood exposure was therefore retained as the climate-exposure measure in the final model. This model-selection result indicates that temperature rise and drought supplied no incremental explanatory information conditional on the retained covariates; it does not imply that these hazards are substantively unimportant in other settings. This comparison was conducted at an intermediate stage of model specification, prior to the full decomposition of the remaining governance and structural covariates (early-warning commitment, adaptation policy, GDP, and agricultural land) into their between- and within-country components; the final specification, presented later in the analysis, reflects that fuller decomposition.

Multicollinearity was assessed using variance inflation factors. VIF values in the final model ranged from 1.008 to 2.253, well below conventional thresholds, indicating that collinearity did not materially affect estimation.

Sensitivity to the operational definition of high food self-sufficiency was evaluated by re-estimating the complete within–between specification using thresholds of 0.75, 0.80, and 0.90 in addition to the primary 0.85 cutoff. The robustness assessment focuses on the stability of coefficient direction, approximate magnitude, and statistical inference across alternative definitions rather than on direct comparison of information criteria across models with different dependent variables. Six theoretically motivated climate-governance interaction terms were also examined in supplementary specifications; none reached conventional statistical significance, and the parsimonious additive model was retained.

As an additional robustness check, the primary 85% specification was re-estimated with year fixed effects to absorb shocks common to all countries in a given year, including global disruptions during the COVID-19 period and international food-market disturbances. The year effects were not jointly significant (likelihood-ratio χ^2^(10) = 2.17, *p* = 0.995), and the principal between-country adaptation-policy association was essentially unchanged (OR = 6.705, 95% CI: 1.494–30.101, *p* = 0.013). The more parsimonious specification without year fixed effects was therefore retained as the primary model.

All analyses were performed using R statistical software (version 4.5.1). Logistic models were estimated using glm with a binomial logit link; country-clustered covariance matrices were calculated using the sandwich and lmtest packages; and variance inflation factors were obtained using the car package.

## 3. Results

### 3.1. Analytical Sample, Panel Variation, and Model Diagnostics

The analytical sample comprised 1111 complete country-year observations for 101 countries observed annually from 2012 through 2022. Table 2 summarizes the overall, between-country, and within-country variation in the study variables. The continuous SSR averaged 0.916 (SD = 0.289), while 64.6% of country-year observations exceeded the primary 0.85 threshold. For most variables, between-country variation was greater than within-country variation, although the extent of temporal variation differed substantially across predictors. Climate-finance flows, risk-management coordination, and formal adaptation-policy presence exhibited meaningful within-country variation (within-country SDs = 20.126, 24.987, and 0.340, respectively). In contrast, commitment to managing exposure and early warning showed no within-country variation and was therefore represented only by its between-country components in the model. Flooding exhibited substantial cross-country variation but negligible within-country variation (between-country SD = 23.220; within-country SD = 0.009). Only 10 of the 101 countries showed any temporal variation in flooding, indicating that its within-country coefficient is identified from a small subset of countries and should therefore be interpreted cautiously. LnGDP and agricultural land also varied predominantly between countries, with relatively limited within-country variation.

The underlying continuous SSR had a mean of 0.916 and a median of 0.920, with an interquartile range of 0.800–1.035 and an observed range of 0.090–2.460. No SSR observations were missing in the analytical sample. Values above 1.0 occurred in 355 country-year observations (32.0% of the sample), reflecting that the ratio is not intrinsically bounded at 100%. At the 75%, 80%, 85%, and 90% self-sufficiency thresholds, respectively, 889, 828, 718, and 605 observations were classified as high self-sufficiency.

The primary binary outcome was highly persistent over time. Of the 101 countries, 57 remained above the 85% SSR threshold throughout 2012–2022, 28 remained below it throughout, and 16 crossed the threshold at least once. Across consecutive country-year observations, 16 upward transitions and 24 downward transitions were observed.

The within–between specification separates persistent cross-country differences from temporal changes within the same country. For a predictor that varies over time, the between-country component is the country-specific mean over 2012–2022, whereas the within-country component is the annual deviation from that country mean. Thus, a between-country coefficient compares countries with different long-run levels of a predictor, while a within-country coefficient captures whether departures from a country’s own long-run level are associated with the probability of high food self-sufficiency.

The temporal-variation check showed substantial differences across predictors (Table 3). Climate-finance flows, risk-management coordination, agricultural adaptation policy, GDP per capita, and agricultural land changed in many countries over the study period, whereas commitment to managing exposure and the early-warning indicator showed no within-country change. Flooding changed in only 10 countries. These diagnostics informed the decomposition used in the final model.

Inference was based on country-clustered standard errors to account for within-country dependence across repeated annual observations. Coefficients are reported as log-odds and odds ratios, with 95% confidence intervals calculated from the country-clustered covariance matrix. Variance inflation factors ranged from 1.008 to 2.253, indicating no evidence of problematic multicollinearity.

### 3.2. Main Within–Between Logistic Regression

Table 4 reports the final pooled logistic model with a within–between decomposition for the primary outcome, SSR > 0.85. Two associations reached the 5% significance level: the between-country component of agricultural adaptation policy and the within-country component of climate-finance flows. All other coefficients were not statistically significant at the 5% level.

**Figure 1:**
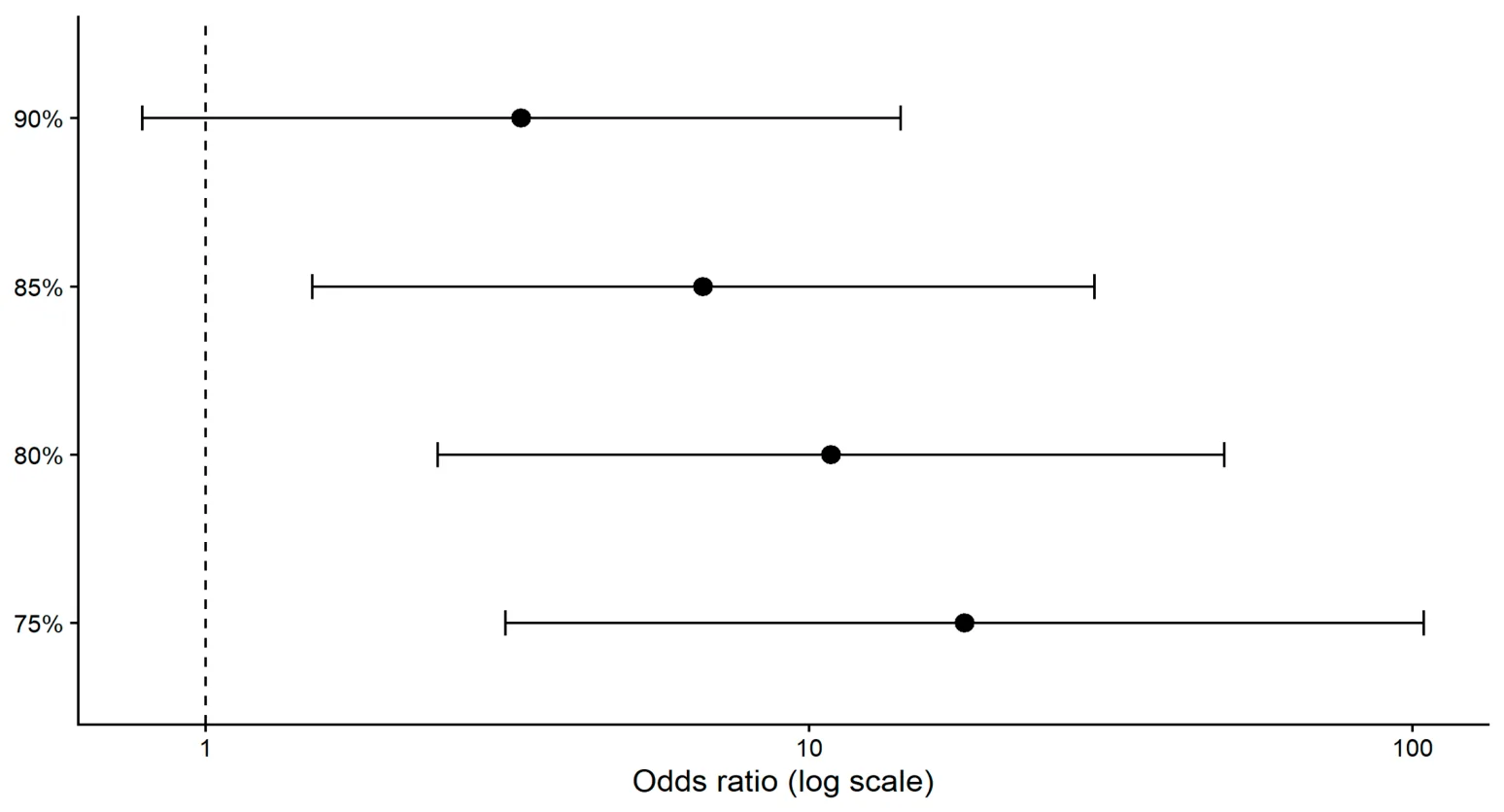
Between-country effect of agricultural adaptation policy on the odds of high SSR, across four alternative thresholds (75%, 80%, 85%, 90%). Note: Points represent odds ratios and horizontal lines represent country-clustered 95% confidence intervals. The odds-ratio axis is shown on a logarithmic scale, and the dashed vertical line indicates an odds ratio of 1.

The clearest positive association concerned the between-country component of agricultural adaptation policy. Countries with a higher long-run prevalence of the coded adaptation-policy indicator had substantially higher odds of exceeding the 85% SSR threshold (β = 1.898, OR = 6.676, *p* = 0.013), as shown in Figure 1. Because the adaptation-policy indicator is binary, its country-level mean represents the proportion of study years in which formal policy presence was recorded. Accordingly, the odds ratio of 6.676 corresponds to a full one-unit difference in this proportion, for example, a comparison between a country with no recorded policy presence during the study period and one with policy presence recorded throughout the period, and should not be interpreted as the effect of a one-year change in policy status. The wide 95% confidence interval (1.502–29.668) indicates substantial uncertainty regarding the magnitude of this association. In the GFSI source framework, this indicator refers to whether a current national climate-change strategy includes adaptation measures for agriculture. The estimate therefore describes a cross-country association with the long-run policy-status measure; it does not demonstrate that adopting a policy causes an immediate increase in self-sufficiency. Average predicted probabilities provide a more interpretable representation of this association. The predicted probability of SSR > 0.85 increased from 0.395 (95% CI: 0.192–0.597) at AdaptPolicy_mean = 0 to 0.490 (0.343–0.638), 0.587 (0.491–0.684), 0.679 (0.597–0.761), and 0.760 (0.658–0.862) at values of 0.25, 0.50, 0.75, and 1.00, respectively. The average probability difference between the 0 and 1 scenarios was 0.366 (95% CI: 0.104–0.627; *p* = 0.006).

The corresponding within-country adaptation-policy coefficient was small and not statistically significant (β = 0.025, *p* = 0.786). Thus, countries that changed their coded adaptation-policy status during the observation period did not show a contemporaneous change in the probability of exceeding the 85% threshold after adjustment for the other covariates. The contrast between the significant between-component and the null within-component indicates that the principal association is cross-sectional rather than a detectable short-run within-country effect. The leave-one-country-out analysis further indicated that the between-country adaptation-policy association was not driven by any single country. The coefficient remained positive and statistically significant in all 101 re-estimated models, with corresponding odds ratios ranging from 5.799 to 9.335 and *p*-values from 0.005 to 0.022.

**Figure 2:**
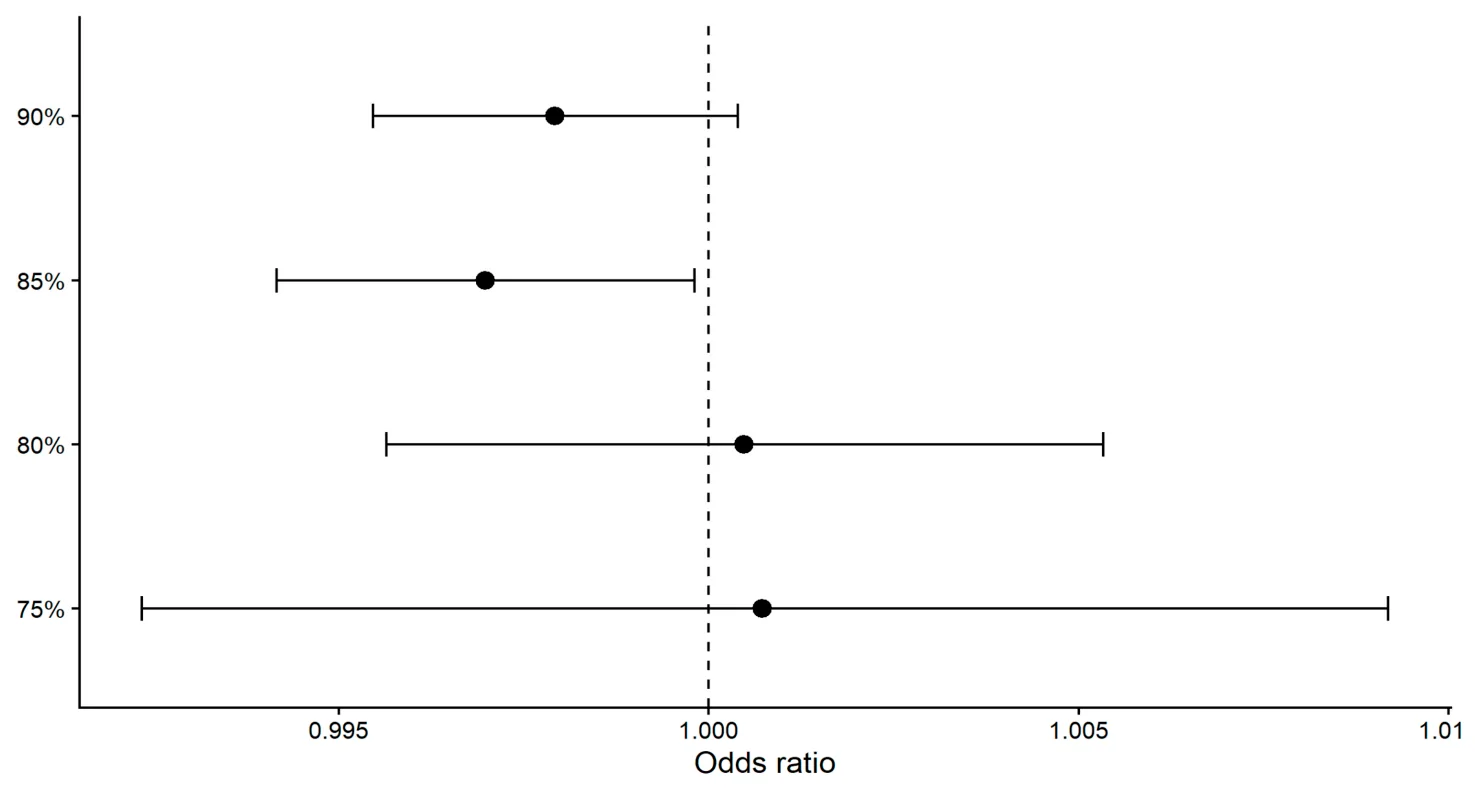
Within-country effect of climate-finance flows on the odds of high SSR, across four alternative thresholds (75%, 80%, 85%, 90%). Note: Points represent odds ratios and horizontal lines represent country-clustered 95% confidence intervals. The dashed vertical line indicates an odds ratio of 1.

The within-country component of climate-finance flows was negative and statistically significant (β = −0.003, OR = 0.997, *p* = 0.036). In years when a country’s harmonized climate-finance value was above its own long-run average, the odds of high self-sufficiency were slightly lower, as presented in Figure 2. This contemporaneous association should not be interpreted causally: climate-related development assistance may increase when adaptation needs or vulnerability are greater, so targeting and reverse causality are plausible explanations. The within-country climate-finance association should be regarded as a secondary and threshold-sensitive finding, as its statistical evidence was not consistent across alternative SSR thresholds.

Neither the between- nor within-country flood coefficients were significant in the primary model (*p* = 0.124 and *p* = 0.875, respectively). Likewise, the between-country components of climate-finance flows, commitment to managing exposure, and risk-management coordination were not independently associated with high SSR. The within-country risk-management coefficient was also not significant (*p* = 0.173).

Early-warning commitment showed no independent association with SSR > 0.85 (*p* = 0.669). Because this indicator was already available in the analytical dataset as a binary measure and showed no within-country change, it is represented only by its country-level mean. Both the between- and within-country GDP coefficients were negative but not significant at the 5% level (*p* = 0.102 and *p* = 0.359). Agricultural land share, entered as a country-level structural component, was positive but imprecisely estimated (*p* = 0.377).

Taken together, the within–between decomposition indicates that the principal association identified in the main model is structural rather than short-term. Countries characterized by a stronger long-run presence of agricultural adaptation policy had substantially higher odds of exceeding the 85% self-sufficiency threshold, whereas within-country changes in adaptation-policy status were not independently associated with contemporaneous changes in high self-sufficiency. Conversely, the significant climate-finance association emerged in the within-country component rather than the between-country component, although this result was less robust across alternative SSR thresholds. For the remaining covariates, the main specification provided limited evidence of systematic within-country associations with high self-sufficiency.

### 3.3. Sensitivity to Alternative SSR Thresholds

The complete final specification was re-estimated using alternative thresholds of 0.75, 0.80, and 0.90, while retaining 0.85 as the primary definition. Table 5 summarizes the coefficients most relevant to the substantive conclusions.

The between-country adaptation-policy association remained positive across all four thresholds, but its magnitude declined as the definition of high self-sufficiency became more stringent: β = 2.896 at 75%, β = 2.386 at 80%, β = 1.899 at 85%, and β = 1.204 at 90%. The association was statistically significant at the 75%, 80%, and 85% thresholds (*p* = 0.001, 0.002, and 0.013, respectively), but not at the 90% threshold (*p* = 0.103). Thus, the direction of the association is consistent across specifications, whereas its magnitude and statistical precision are sensitive to the threshold used (Figure 1). The within-country adaptation-policy term was not statistically significant at any threshold.

This pattern reinforces the interpretation that the observed adaptation-policy relationship is predominantly a persistent between-country association rather than a short-run within-country effect.

Long-run flood exposure was negatively associated with high self-sufficiency at the 75% and 80% thresholds (*p* = 0.004 and *p* = 0.011), but not at 85% or 90%. The within-country climate-finance coefficient was significant only in the 85% specification and was marginal at 90% (*p* = 0.099), indicating that this result is sensitive to the outcome definition. Early-warning commitment and the between-country GDP component were negative and significant at the 75% and 80% thresholds, but neither association persisted at the higher thresholds. The within-country GDP term was marginal only at the 90% threshold (*p* = 0.057).

Overall, the sensitivity analysis identifies the between-country adaptation-policy association as the most consistent result: its coefficient remained positive at all four thresholds and statistically significant at the 75%, 80%, and 85% cutoffs. The associations involving flood exposure, early-warning commitment, GDP, and within-country climate-finance variation were more threshold-dependent and should therefore be interpreted as secondary, specification-sensitive findings rather than universal determinants of food self-sufficiency.

## 4. Discussion and Conclusions

This study examined how climatic exposure, adaptation governance, climate-related finance, and structural national characteristics are associated with the likelihood of attaining high food self-sufficiency, while distinguishing persistent cross-country differences from changes occurring within countries over time. The central empirical pattern is that the strongest evidence concerns enduring institutional differences rather than short-run policy movements. In particular, the sustained presence of national agricultural adaptation policy emerges as the most consistent positive correlate of high SSR.

Countries characterized by a more persistent presence of an up-to-date national climate strategy that includes adaptation measures for agriculture were substantially more likely to exceed the 85% SSR threshold, even after accounting for flood exposure, climate finance, risk-management coordination, GDP, and agricultural land. The association remained positive across all alternative thresholds and statistically significant at 75%, 80%, and 85%. This pattern is consistent with a broader literature emphasizing that effective adaptation depends on institutional continuity, policy coherence, and the integration of climate and agricultural planning rather than on isolated technical measures [6,10,11,16,24]. Persistent adaptation-policy presence may therefore be associated with broader institutional and planning characteristics that are not directly measured by the indicator.

The distinction between the between-country and within-country components of adaptation policy is critical. Although the long-run policy component was strongly associated with high SSR, the within-country component was not. Thus, countries in which agricultural adaptation policy was present more consistently differed systematically from countries in which it was less consistently present, but a change in policy status within a given country was not associated with an immediate contemporaneous change in high-SSR status. This cautions against interpreting the policy coefficient as a short-run causal effect. Possible explanations for this pattern include delayed relationships with investment, agricultural research, extension services, infrastructure, water management, or farmer behavior, as well as broader unobserved institutional differences between countries; however, these mechanisms were not examined empirically in the present study. Moreover, the GFSI indicator captures formal policy presence rather than implementation quality, budget execution, enforcement, or farm-level uptake. The evidence therefore supports a narrower claim: sustained formal integration of agricultural adaptation into national climate policy is associated with a greater likelihood of high food self-sufficiency.

Climate-finance flows reveal a different temporal pattern. Their between-country component was not statistically significant in the main specification, whereas the within-country component was negative and significant at the 85% threshold. This means that the relevant association concerns years in which a country’s climate-finance score was above its own longer-run average, rather than persistent differences between recipient countries. The result should not be interpreted as evidence that climate finance reduces self-sufficiency. Climate-related development assistance is not randomly allocated and may increase precisely where exposure, institutional constraints, or adaptation needs are greatest. In addition, financing is unlikely to affect agricultural production contemporaneously because project preparation, infrastructure investment, institutional reform, and technological diffusion typically operate with lags. The sensitivity analysis reinforces this caution: the within-country finance association was significant at 85%, marginal at 90%, and absent at 75% and 80%. It is therefore best treated as a secondary, threshold-sensitive finding that may reflect targeting or timing rather than a harmful effect of finance.

Flood exposure and GDP also produced threshold-dependent results. Persistent flood exposure was negatively associated with the probability of exceeding the 75% and 80% SSR thresholds, but not the 85% or 90% thresholds. Flooding can constrain agricultural production through crop losses, soil erosion, infrastructure damage, and disruption of storage and transport [3,4], yet the indicator used here largely captures projected structural exposure and changed within relatively few countries. The analysis is therefore more informative about long-run differences in vulnerability than about short-run flood shocks. GDP per capita was similarly negative at the lower thresholds but not significant in the main 85% specification or at 90%. A plausible interpretation is that wealthier countries can rely more extensively on international markets and comparative advantage rather than maximizing domestic production, consistent with the distinction between food self-sufficiency and food security emphasized by Clapp [13] and comparative evidence reported by Barel-Shaked and Buda [15]. Because these associations vary across thresholds, neither should be treated as a universal determinant of high SSR.

Agricultural land share was positive but imprecisely estimated, and adding a within-country land component did not improve model fit. Land availability therefore appears to represent productive potential rather than an automatic determinant of high self-sufficiency. Its productive value depends on land quality, water, technology, input use, crop composition, climate conditions, and management practices [3,18,19]. Other governance indicators also lacked robust independent associations in the main specification. Commitment to managing exposure and risk-management coordination were not significant at the 85% threshold, while early-warning commitment was significant only at lower thresholds. These results underscore that governance indicators capture distinct institutional processes and should not be treated as interchangeable measures of adaptation capacity. Some reflect formal commitments, others may intensify in response to vulnerability, and some may affect agricultural outcomes only after substantial lags.

Taken together, the within–between decomposition provides the study’s main substantive insight: the evidence is stronger for persistent structural and institutional differences across countries than for contemporaneous within-country changes. This is especially clear for agricultural adaptation policy. The finding does not imply that within-country reform is ineffective; rather, it suggests that institutional effects may accumulate gradually and that annual indicators may not capture implementation. More broadly, the results should not be read as an argument for food autarky or for maximizing SSR in every country. Food self-sufficiency is distinct from food security, and international trade can enhance availability, diversity, and stability where domestic production faces ecological or resource constraints [13,14]. The policy implication is narrower: where maintaining domestic productive capacity is considered strategically important, durable adaptation strategies may form part of the institutional foundation needed to sustain that capacity under climate pressure, but only when translated into implementation, investment, risk information, infrastructure, research, and coordination.

Several limitations qualify these conclusions. First, the SSR used in this study is an aggregate quantity-based measure constructed from national production and trade data rather than a complete commodity-level food-balance account. Aggregating heterogeneous food-item categories in physical quantities may obscure differences in commodity composition and may introduce aggregation overlap where broad product groups and more disaggregated items coexist in the source data. In addition, the measure does not separately account for stock variation, feed, seed, losses, processing, or other non-food uses. It therefore should be interpreted as an operational indicator of aggregate domestic-production reliance rather than as a precise measure of food available for final human consumption, and it does not capture nutritional composition, commodity-specific dependence, affordability, or distributional access.

Second, although the GFSI indicators enable systematic cross-country comparison, they simplify institutional conditions and do not directly measure policy implementation, enforcement, or subnational variation. Third, endogeneity and reverse causality remain important concerns, as climate finance, risk-management activity, and policy adoption may themselves respond to climatic vulnerability or agricultural stress. Persistent between-country associations may also reflect unobserved structural differences between countries that are not fully captured by the included covariates. The within–between decomposition distinguishes persistent cross-country differences from within-country change, while country-clustered standard errors account for dependence among repeated observations; neither provides causal identification. Fourth, dichotomizing SSR necessarily reduces information contained in the underlying continuous measure. Although the binary specification corresponds to the study’s substantive focus on comparatively high self-sufficiency, the sensitivity analyses show that the magnitude and statistical precision of the adaptation-policy association vary with the selected threshold, even though its direction remains positive across all four cutoffs. Finally, the national-level design cannot capture potentially important subnational heterogeneity in climatic exposure, agricultural production, or policy implementation.

Despite these limitations, the study contributes to the climate-food literature by integrating climatic exposure, adaptation governance, climate finance, economic structure, and agricultural resources while explicitly separating persistent between-country differences from within-country changes. This distinction is especially important in global governance research because many institutional indicators are stable or slowly changing. Treating them as ordinary annual covariates risks conflating enduring institutional differences with short-run policy variation.

The principal conclusion is therefore both substantive and methodological. High national food self-sufficiency appears to be associated less with short-term movements in individual governance indicators than with persistent national institutional conditions. Among the factors examined, sustained agricultural adaptation policy is the most consistent positive correlate of high SSR, while within-country changes in policy status show no contemporaneous association. This pattern is consistent with an association between persistent formal policy presence and high SSR, although the indicator does not capture policy implementation, enforcement, quality, or institutional embedding. For policymakers, the implication is not that formal strategies automatically generate self-sufficiency, nor that domestic production should replace trade-based food strategies. Rather, the findings identify a persistent association between formal agricultural adaptation-policy presence and high SSR; whether strengthening, implementing, or sustaining such policies leads to changes in domestic production capacity requires further causal investigation. Future research should move beyond policy presence toward implementation, timing, financing, and commodity- and subnational-level outcomes in order to determine whether the persistent institutional associations identified here translate into causal improvements in domestic production capacity under a changing climate.

## Figures and Tables

**Table 1:** Variables used in the empirical analysis.

Variable	Definition and Analytical Coding	Scale/Unit	Source	Role in Model
High food self-sufficiency (SSR_D85)	Binary outcome equal to 1 when SSR exceeds 0.85 and 0 otherwise. SSR is calculated as production divided by production plus imports minus exports; the percentage formulation is multiplied by 100.	SSR: percentage in the source formulation; proportional form (0–1) in the analytical dataset. Outcome: binary (0/1).	FAO	Dependent variable
Flooding	Projected percentage change in flood hazard from 1960–1990 to 2040–2070 under RCP 4.5; flood hazard is based on the monthly maximum precipitation over five consecutive days.	Original GFSI unit: % change in flood hazard; analytical GFSI score: 0–100.	GFSI-Sustainability & Adaptation	Climate exposure; between and within components
Climate finance flows	Climate mitigation- and adaptation-related official development assistance allocated to recipient countries.	Original GFSI unit: US$ per capita (2019 dollars); analytical GFSI score: 0–100.	GFSI-Sustainability & Adaptation	Climate finance; between and within components
Commitment to managing exposure	Assessment of whether agriculture-related climate exposure and natural-resource management are addressed under Nationally Determined Contributions (NDCs).	Original GFSI rating: 0–13; analytical GFSI score: 0–100.	GFSI-Sustainability & Adaptation	Institutional capacity; between component
Risk management coordination	Whether disaster-risk management and adaptation/mitigation measures are coordinated, based on the share of local governments implementing local disaster-risk-reduction strategies aligned with national strategies.	Original GFSI unit: percentage; analytical GFSI score: 0–100.	GFSI-Sustainability & Adaptation	Institutional coordination; between and within components
Early-warning commitment (EarlyWarning_D)	GFSI indicator of whether the country committed to agricultural early-warning measures and invested in climate-smart agricultural practices. The variable was available as a binary indicator in the analytical dataset.	Original GFSI rating: 0–2 (2 = best); analytical variable: binary (0/1).	GFSI-Sustainability & Adaptation	Policy preparedness
Agricultural adaptation policy (AdaptPolicy_D)	GFSI indicator of whether the country has a current national climate-change strategy covering adaptation for agriculture. The variable was available as a binary indicator in the analytical dataset.	Original GFSI rating: 0–2 (2 = best); analytical variable: binary (0/1).	GFSI-Sustainability & Adaptation	Adaptation policy
GDP per capita (Ln_GDP)	Natural logarithm of GDP per capita adjusted for purchasing power parity at constant 2015 prices.	Natural log.	FAO	Economic control
Agricultural land (LAND)	Share of total national land area classified as agricultural land.	Original FAOSTAT unit: % of land area; proportion (0–1) in the analytical dataset.	FAOSTAT	Structural agricultural control; between component only

Note: For GFSI indicators, the table distinguishes the original GFSI measurement scale from the harmonized scale contained in the analytical dataset. Early-warning commitment and agricultural adaptation policy were already available as binary variables in the analytical file; no unverified recoding rule is imposed here. Agricultural land is sourced from FAOSTAT and is expressed as a proportion (0–1) in the analytical dataset.

**Table 2:** Descriptive statistics and within- and between-country variation.

Variable	Mean	SD	Min	Max	Between-Country SD	Within-Country SD
SSR	0.916	0.289	0.09	2.46	0.285	0.052
SSR_D85	0.646	0.478	0	1	0.454	0.157
Flooding	65.985	23.116	0	100	23.22	0.009
Climate finance flows	41.493	40.505	0	100	35.31	20.126
Commitment to managing exposure	40.596	25.843	0	76.9	25.96	0
Risk-management coordination	22.509	38.093	0	100	28.884	24.987
Early warning	0.574	0.495	0	1	0.497	0
Adaptation-policy presence	0.673	0.469	0	1	0.325	0.34
LnGDP	8.621	1.501	5.568	11.506	1.504	0.105
Land	0.427	0.203	0.027	0.828	0.203	0.011

**Table 3:** Within-country temporal variation in explanatory variables.

Variable	Countries with Within-Country Change (N = 101)
Flooding	10
Climate finance flows	74
Commitment to managing exposure	0
Risk-management coordination	60
Early-warning commitment	0
Agricultural adaptation policy	65
GDP per capita (Ln_GDP)	101
Agricultural land (LAND)	95

Note: A variable is counted as changing within a country when it takes more than one observed value during 2012–2022. The table describes whether any within-country change occurred; it does not measure the magnitude of that change.

**Table 4:** Within–between logistic regression predicting high food self-sufficiency (SSR > 0.85).

Predictor	β	Clustered SE	OR	95% CI for OR	*p*
Flooding: between	−0.018	0.012	0.982	0.959–1.005	0.124
Flooding: within	0.068	0.431	1.070	0.460–2.489	0.875
Climate-finance flows: between	−0.013	0.009	0.987	0.970–1.004	0.136
Climate-finance flows: within	−0.003	0.001	0.997	0.994–1.000	0.036 *
Commitment to managing exposure: between	0.009	0.012	1.009	0.987–1.032	0.436
Risk-management coordination: between	0.004	0.008	1.004	0.988–1.020	0.631
Risk-management coordination: within	−0.002	0.001	0.998	0.995–1.001	0.173
Early-warning commitment: between	−0.228	0.532	0.796	0.281–2.259	0.669
Agricultural adaptation policy: between	1.898	0.761	6.676	1.502–29.668	0.013 *
Agricultural adaptation policy: within	0.025	0.094	1.026	0.854–1.232	0.786
GDP per capita (Ln_GDP): between	−0.302	0.184	0.740	0.515–1.061	0.102
GDP per capita (Ln_GDP): within	−0.416	0.454	0.660	0.271–1.605	0.359
Agricultural land (LAND): between	1.014	1.149	2.757	0.290–26.193	0.377

* *p* < 0.05. Note: Observations: 1111; Countries: 101; Outcome events (SSR > 0.85): 718; Log-likelihood: −607.945; AIC: 1243.890; BIC: 1314.072.

**Table 5:** Sensitivity analysis using alternative definitions of high food self-sufficiency.

Predictor	SSR > 0.75	SSR > 0.80	SSR > 0.85	SSR > 0.90
Flooding: between	−0.045 [−0.075, −0.015]	−0.035 [−0.062, −0.008]	−0.018 [−0.042, 0.005]	−0.013 [−0.034, 0.007]
Climate-finance flows: within	0.001 [−0.008, 0.009]	0.000 [−0.004, 0.005]	−0.003 [−0.006, −0.0002]	−0.002 [−0.005, 0.0004]
Early-warning commitment: between	−2.017 [−3.357, −0.677]	−1.298 [−2.504, −0.093]	−0.228 [−1.270, 0.815]	−0.029 [−1.027, 0.969]
Agricultural adaptation policy: between	2.896 [1.144, 4.648]	2.386 [0.885, 3.886]	1.898 [0.407, 3.390]	1.204 [−0.242, 2.650]
Agricultural adaptation policy: within	0.035 [−0.116, 0.185]	−0.006 [−0.129, 0.118]	0.025 [−0.158, 0.209]	0.042 [−0.182, 0.267]
GDP per capita (Ln_GDP): between	−0.895 [−1.501, −0.288]	−0.667 [−1.115, −0.220]	−0.302 [−0.663, 0.059]	−0.109 [−0.441, 0.223]
GDP per capita (Ln_GDP): within	−2.370 [−5.464, 0.723]	−0.285 [−1.709, 1.140]	−0.416 [−1.305, 0.473]	−0.925 [−1.878, 0.028]

Note: Entries are logit coefficients with country-clustered 95% confidence intervals in brackets.

## Data Availability

Data is contained within the article or Supplementary Material. The original contributions presented in this study are included in the article/Supplementary Material. The processed country-year analytical dataset used in this study, comprising 1111 observations from 101 countries over 2012–2022, is provided in the Supplementary Materials. The R replication script used to reproduce the primary model and the reported robustness and sensitivity analyses is also provided.

## Supplementary Materials

The following supporting information can be downloaded at: https://www.mdpi.com/article/10.3390/foods15193492/s1: Table S1, Countries included in the final analytical sample; the dataset used for the empirical analyses; and the R code used to replicate the analyses reported in the manuscript.
